# Integrating qualitative research in a multi-centre trial - the clinical trials unit perspective

**DOI:** 10.1186/1745-6215-14-S1-P67

**Published:** 2013-11-29

**Authors:** CA Rogers, G Mazza, S Paramasivan, N Smith, R Nash, JM Blazeby, J Donovan

**Affiliations:** 1 Clinical Trials and Evaluation Unit, School of Clinical Sciences, University of Bristol, Bristol, UK; 2 School of Social and Community Medicine, University of Bristol, Bristol, UK

## Background

Recruitment into surgical trials is often hampered because of surgeon or patient preferences. Qualitative research to explore and standardise methods for communicating clinical equipoise and addressing patient concerns has been shown to be effective in maximising recruitment. Often this research runs alongside the trial without being fully integrated within it. We describe how we integrated qualitative research into the multi-centre By-Band study comparing two surgical procedures for morbid obesity.

## Methods

The integration has the following elements (a) having one screening log which captures patient consent for i) the qualitative research, ii) simple follow up concerning treatment preference, and iii) the randomised trial; (b) capturing details of the audio-recordings taken during the consultation process within the participant case report form; (c) adding functionally to the trial database to capture this information and to allow secure up-load of audio-recordings directly into the database; (d) producing routine reports for the qualitative researchers.

## Results

Participating sites have found the system straightforward to use. The database is hosted on an NHS server accessed via the NHS clinical portal, so is familiar to centres taking part. Audio-recordings are being received in a timely way and real-time queries in the database have led to high levels of completeness. Up-to-date information on trial progress is provided to the qualitative researchers on a weekly basis, with minimal effort.

## Conclusion

Integrating the qualitative research fully into the trial has brought benefits for all. The mechanism for transferring audio-recordings is being rolled out to other trials with a qualitative component.

## Disclaimer

This study is funded by the NIHR HTA programme and the views and opinions expressed are those of the authors and do not necessarily reflect those of the HTA programme and, NIHR, NHS or the department of health.

